# Omega-3 fatty acids in bipolar patients with a low omega-3 index and reduced heart rate variability: the “BIPO-3” trial

**DOI:** 10.1186/s40345-022-00253-9

**Published:** 2022-04-01

**Authors:** Michael Berger, Florian Seemüller, Alessandra Voggt, Michael Obermeier, Franca Kirchberg, Anja Löw, Michael Riedel, Clemens von Schacky, Emanuel Severus

**Affiliations:** 1 Zeughaus Practice, Zurich, Switzerland; 2 Department of Psychiatry and Psychotherapy, Ludwig-Maximilians-Universität München, Munich, Germany; 3 Department of Psychiatry, Psychosomatic and Psychotherapy, Kbo-Lech-Mangfall-Clinic Garmisch-Partenkirchen, Garmisch-Partenkirchen, Germany; 4 St. Joseph Krankenhaus, Klinik Für Seelische Gesundheit Im Kindes- Und Jugendalter, Berlin, Germany; 5 Gesellschaft Für Therapieforschung mbH, Munich, Germany; 6 Division of Metabolic and Nutritional Medicine, Dr. Von Hauner Children’s Hospital, Ludwig-Maximilians-Universität München, Munich, Germany; 7 Department of Internal Medicine I – Cardiology, Ludwig-Maximilians-Universität München, Munich, Germany; 8 Marion Von Tessin Memory-Zentrum gGmbH, Munich, Germany; 9 Department of Preventive Cardiology, Ludwig- Maximilians-Universität München, Munich, Germany; 10 Omegametrix, GmbH, Planegg, Germany; 11 Department of Psychiatry and Psychotherapy, TU Dresden, Dresden, Germany

**Keywords:** Bipolar disorders, Omega-3 fatty acids, Heart rate variability, omega-3 index, Randomised controlled trial

## Abstract

**Background:**

Research suggests that a low omega-3 index may contribute to the low heart rate variability and the increased risk of cardiovascular morbidity and mortality in bipolar disorders. However, so far, no intervention trial with EPA and DHA has been conducted in bipolar patients attempting to increase their heart rate variability.

**Methods:**

119 patients with bipolar disorder according to DSM-IV were screened, with 55 euthymic bipolar patients—owing to inclusion criteria (e.g. low omega-3 index (< 6%), SDNN < 60 ms.)—being enrolled in a randomized, double-blind, 12-week parallel study design with omega-3 fatty acids (4 capsules of 530 mg EPA, 150 mg DHA) or corn oil as a placebo, in addition to usual treatment. Heart rate variability as well as the omega-3 index were measured at baseline and at the endpoint of the study.

**Results:**

A total of 42 patients (omega-3: n = 23, corn oil: n = 19) successfully completed the study after 12 weeks. There was a significant increase in the omega-3 index (value at endpoint minus value at baseline) in the omega-3 group compared to the corn oil group (p < 0.0001). However, there was no significant difference in the change of the SDNN (value at endpoint minus value at baseline) between the treatment groups (p = 0.22). In addition, no correlation between changes in SDNN and change in the omega-3 index could be detected in the omega-3 group (correlation coefficient = 0.02, p = 0.94) or the corn oil group (correlation coefficient =  − 0.11, p = 0.91). Similarly, no significant differences between corn oil and omega-3 group regarding the change of LF (p = 0.19), HF (p = 0.34) and LF/HF ratio (p = 0.84) could be demonstrated.

**Conclusions:**

In our randomized, controlled intervention trial in euthymic bipolar patients with a low omega-3 index and reduced heart rate variability no significant effect of omega-3 fatty acids on SDNN or frequency-domain measures HF, LF and LF/HF ratio could be detected. Possible reasons include, among others, the effect of psychotropic medication present in our trial and/or the genetics of bipolar disorder itself. Further research is needed to test these hypotheses.

*Trial registration* ClinicalTrials.gov, NCT00891826. Registered 01 May 2009–Retrospectively registered, https://clinicaltrials.gov/ct2/show/NCT00891826

## Introduction

Bipolar disorders are common diseases, with a lifetime prevalence of around 1–5%, depending on the definition applied (Merikangas et al. 2011) and are associated with substantial disability (Vos et al. 2012) and reduced life expectancy Kessing et al. 2015a, b). Most studies suggest that patients with bipolar disorders are at increased risk of cardiovascular morbidity and mortality (Goldstein et al. 2015a, b; Marshe al. 2017; Prieto et al. 2014; Wulsin et al. 2018). A low heart rate variability (HRV) is thought to be a risk factor for cardiovascular morbidity and mortality (Huikuri and Stein 2013), specifically in patients with affective disorders and comorbid heart disease (Carney et al. 2005). Even when being euthymic, i.e. showing no significant symptoms, a substantial proportion of bipolar patients, possibly in particular those with more advanced stages of the disorder (Freyberg et al. 2020), have decreased heart rate variability compared to a control group, irrespective of specific pharmacological treatment, possibly indicating a shift of sympathovagal balance towards vagal tone predominance and a reduced sympathetic tone (Cohen et al. 2003). The reasons for this are still largely unclear (Drewery et al. 2017; Faurholt-Jepsen et al. 2017). Notwithstanding, the available data suggest that reduced heart rate variability could therefore contribute to the increased cardiovascular morbidity and mortality in patients with bipolar disorder.

In bipolar patients, levels of the two omega-3 fatty acids eicosapentaenoic acid (EPA) and docosahexaenoic acid (DHA) have been found to be low in most (Faurholt-Jepsen et al. 2017; Freyberg et al. 2020), but not all cross-sectional studies (Voggt et al. 2015), including studies with individuals at risk of or with first-episode bipolar disorder (McNamara et al. 2015, 2016; Wulsin et al. 2018). Meta-analyses have demonstrated that EPA-predominant formulations improve symptoms of clinically diagnosed depression (Liao et al. 2019; Saunders et al. 2016), which prompted guidelines to adopt this approach (Guu et al. 2019). Furthermore, omega-3 fatty acids have been demonstrated to increase heart rate variability in many intervention trials in different patient populations (Rovere and Christensen 2015) and may reduce the risk of coronary death and coronary events (Abdelhamid et al. 2020; Zelniker et al. 2021). However, so far, no intervention trial with EPA and DHA has been conducted in bipolar patients attempting to increase their heart rate variability. Therefore, we tested the hypothesis that omega-3 fatty acids significantly improve heart rate variability (Severus et al. 1999), measured as standard deviation of the normal-to-normal interval (SDNN, ms), in a randomized, double blind controlled intervention trial in euthymic patients with bipolar disorders with a low omega-3 index (Harris and von Schacky 2004, 2007) and reduced heart rate variability.

## Methods

### Participant recruitment

Potential trial participants were screened in the inpatient and outpatient units of the Department of Psychiatry and Psychotherapy of the Ludwig-Maximilians-University, Munich. Potentially eligible patients were approached by one of the authors (MB) and informed about the study. If patients were interested in participating in this study, the following clinical inclusion and exclusion criteria were checked. Patients who were (1) diagnosed with bipolar disorders (I, II) in remission according to the Structured Clinical Interview (SCID) for the Diagnostic and Statistical Manual of Mental Disorders Fourth Edition (DSM-IV), (2) able to give written informed consent, (3) between 18 and 65 years of age, (4) on stable psychotropic medication for at least 2 weeks, (5) fluent in German or English to complete baseline and follow-up interviews met the clinical inclusion criteria. Clinical exclusion criteria were (1) a diagnosis of current substance abuse (with or without substance dependence), (2) intake of omega-3 fatty acids was indicated according to recent treatment guidelines, (3) treatment with anticoagulants, (4) any acute or life-threatening comorbidity, such as collapse and shock, acute myocardial infarction, stroke, embolism, or disease seriously limiting life expectancy (5) current significant suicidal or homicidal risk in the investigator’s judgement, (6) low likelihood of compliance with the study protocol, (7) childbearing potential without a medically accepted method of contraception, pregnancy or breastfeeding.

If patients were eligible, they were asked to sign a written informed consent form. After signing, diagnosis was confirmed using the structured clinical interview for DSM-IV (Wittchen et al. 1997). A blood sample was drawn for determination of the omega-3 Index and heart rate variability was measured. A low omega-3-index (< 6%), and a low SDNN (< 60 ms) were inclusion criteria numbers 6 and 7. Patients fulfilling all inclusion and no exclusion criteria were recruited for the trial.

The present trial was approved by the ethics’ committee of the medical faculty of the Ludwig-Maximilians-University, Munich, registered at Clinicaltrials.gov (NCT00891826), and conducted between January 2009 and April 2012 according to the Guidelines laid down in the Declaration of Helsinki and Good Clinical Practice. Informed consent allowed analysis of all the clinical and laboratory data mentioned in the present report. The trial was initiated, designed, conducted, and evaluated by the investigators, and the sponsor had no role in study design, data acquisition, or evaluation or preparation of the manuscript.

### Trial design

The present trial was a randomized, double-blind, single-center, 12-week parallel study comparison of omega-3 fatty acids vs. corn oil, in addition to usual treatment. The primary endpoint of the trial was a change in HRV, as assessed by SDNN in ms. Predefined secondary endpoints were a change in HRV, as assessed by a ratio of low frequency to high frequency (LH/HF ratio); new episodes of bipolar depression; and mood rating scales.

### Procedures

Eligible patients were randomized to 4 capsules EPAX 6015 TG per day (2 in the morning, 2 in the evening), each containing 530 mg of EPA (eicosapentaenoic acid) and 150 mg of DHA (docosahexaenoic acid) as triglycerides or 4 matching capsules containing corn oil as placebo, to be taken with a meal to maximize bioavailability. Both products were produced and provided by EPAX AS: http://www.epax.com/. The placebo was matched to the study drug for taste, color and size. Patients were to continue with their pre-existing psychotropic medication, with adjustments as clinically indicated.

At baseline, demographics, clinical history and medication were assessed by means of the Network Enrolment Questionnaire as previously used by the Stanley Foundation Bipolar Network (Suppes et al. 2001). At baseline and at 12 weeks, HRV and the omega-3 Index and other blood parameters were measured, as was the psychopathological state using standardized rating scales: Young Mania Rating Scale (YMRS), Hamilton Rating Scale for Depression, [HAMD (Hamilton 1967)], Montgomery-Åsberg Depression Rating Scale [MADRS (Montgomery and Asberg 1979)], Beck Depression Inventory [BDI (Beck et al. 1961)] and Clinical Global Impressions Scale for Bipolar Illness [CGI (Spearing et al. 1997)].

### Heart rate variability

HRV was assessed as recently described in more detail (Voggt et al. 2015). A slightly darkened room was used which had a comfortable room temperature. Participants were asked to relax and stay awake during the test period. Careful considerations were made to ensure subjects were not disturbed by noise. Recordings took place at the same time of the day, commonly between 10 am and 2 pm, with few exceptions being equally distributed between verum and placebo groups. A ProSciCard III (CPS medical, Tyler, TX, USA), was used to continuously record electrocardiograms (ECG) in a supine position, during normal breathing, after a short rest during a 30 min interval. The RecordProSciCard computer system (ProSciCard III) was installed for analysing HRV. By using the recorded NN intervals, the standard deviation of the NN interval (SDNN) (as a statistical time domain measure) was calculated (CPS GmbH 2009). The system’s intern check of the data was performed by Task Force Analysis, artefacts were marked. Before elimination of the artefacts, it was double-checked if the artefacts set by the software were correct and if overlooked by the software artefacts could be marked by the investigator (CPS GmbH 2009). Artefacts were defined as a fluctuation range of more than 15% of the RR-Intervals. Using power spectrum analysis frequency domain parameters of HRV were derived with high-frequency power (HFP; defined as 0·15–0·40 Hz) and low-frequency power (LFP; defined as 0·04–0·15 Hz) expressed in normalized units adjusting for changes in total power (which is related to HR).

### Omega-3 index

Erythrocyte fatty acid composition was analysed according to the HS-Omega-3 Index® methodology as previously described (Harris and von Schacky 2004). Results are given as EPA plus DHA expressed as a percentage of total identified fatty acids after response factor correction. The coefficient of variation for EPA plus DHA was 5%. Analyses were quality-controlled according to DIN ISO 15189 (Rovere and Christensen 2015).

### Statistical analyses

The power calculation is based on Cohen et al. (2003), in combination with our own data, the statement of the taskforce (Heart rate variability 1996) and the usual assumptions (alpha = 5%, power = 80%): the primary outcome parameter was defined as standard deviation of all normal RR intervals (SDNN, in ms.). Based on the assumption that SDNN will increase by 10 ms on average in the omega-3 fatty acid group, 23 patients per group were needed; a total of 46 patients. Furthermore, as we expected approximately 10% of our patients to drop out prematurely due to a variety of reasons, we planned to recruit a total number of 51 patients.

Data analysis was carried out using the statistical program R 2.9.0 (Hornik 2012). For categorical data, Fisher's Exact test was used, and Wilcoxon rank sum test for metric variables. In the case of HRV, baseline and endpoint values of SDNN, low frequency (LF), high frequency (HF), LF/HF ratio were compared both within and between groups.

Linear mixed models with random intercept were calculated unadjusted, and adjusted for age and gender, as it has been shown in previous studies that HRV measures decline with advancing age (Bigger et al. 1995; Liao et al. 1995; Zulfiqar et al. 2010), and supplementation with omega-3 fatty acids seems to have a beneficial effect on HRV especially in men (Christensen and Schmidt 2007). Linear regression models that explain heart rate variability (SDNN, LF, HF, LF/HF ratio) best were created: In order to explain the dependent variable group affiliation and omega-3 index were integrated as independent variables. As a next step the change/difference (value at endpoint minus value at baseline) was illustrated. This was calculated for SDNN, omega-3 index, LF, HF and LF/HF ratio. First univariate tests, using Wilcoxon signed-rank test, on differences between placebo and verum group were performed. In addition, unadjusted and adjusted (age, gender) linear mixed models on the changes of SDNN were calculated with group affiliation and change in omega-3 index as independent variables. Furthermore, Pearson correlation coefficients with changes in SDNN were calculated for the change of omega-3 index. For all statistical calculations the significance level was set 5% (p < 0.05).

Finally, a linear regression model within the patient group taking omega-3 as well as the control group taking corn oil was calculated in order to explain SDNN change over time. Explaining variables were EPA change, DHA change and omega-3 index at baseline. Age, gender and diagnosis of bipolar disorder were further co-variables in the model.

## Results

### Study population

**Fig. 1:**
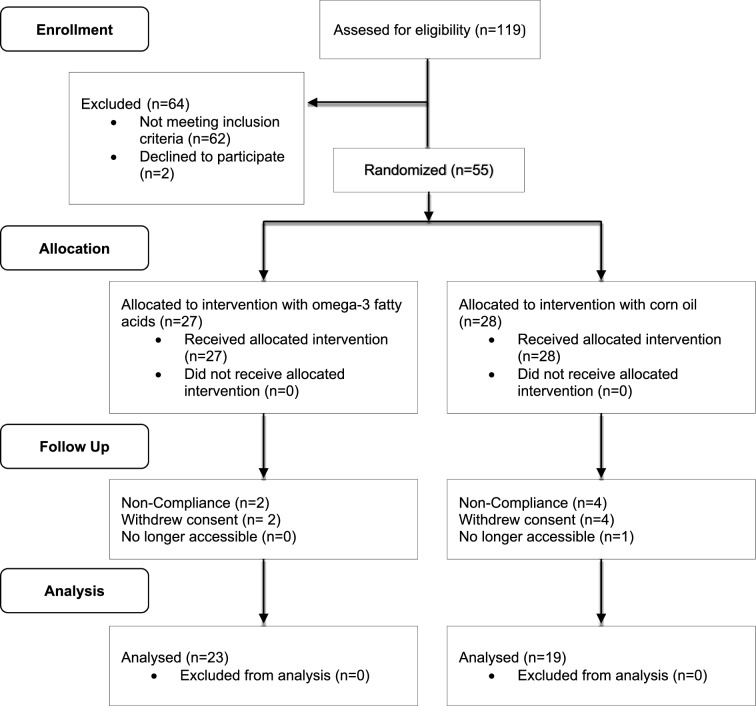
CONSORT Flow Diagram: progress of all participants through the trial

Of 119 patients with Bipolar I/II Disorder screened, 55 patients met the inclusion criteria, and were willing to participate. Of those, 27 were randomized to omega-3 fatty acids, and 28 patients to corn oil. A total of 42 patients (omega-3 fatty acids: n = 23, corn oil: n = 19) completed the study, while 13 did not (omega-3 fatty acids: n = 4, corn oil: n = 9). The reasons were as follows: 6 patients were excluded from the study due to non-adherence to the study protocol, 6 patients withdrew consent, 1 patient was no longer accessible (Fig. 1: Flow Diagram). Demographic and clinical characteristics of study completers are shown in Table 1. No statistical significant differences could be demonstrated.

**Table 1:** Demographic and clinical variables of the study population: mean ± sd [missing values]

	Omega-3 (n = 23)	Corn oil (n = 19)	p-value
Age (years)	46.6 ± 13.25	42.1 ± 10.75	0.33
Gender (m/f)	11/12	8/11	0.76
Bipolar (I/II)	12/5	8/5	0.71
Bipolar NOS	6	6	0.74
Age at onset depression (years)	22.7 ± 10.25	24.2 ± 6.75	0.62
Age at onset mania (years)	29.0 ± 18.00	25.1 ± 7.25	0.36
Number depressive episodes	9.5 ± 7.00	9.9 ± 9.00	0.74
Number manic episodes	5.0 ± 4.00	8.3 ± 4.00	0.86
Episodes mania/depression	0.2 ± 0.25	0.2 ± 0.00	0.76
Hospitalisation depression	2.1 ± 1.75	2.9 ± 1.00	0.77
Hospitalisation mania	1.4 ± 2.00	1. 5 ± 2.00	0.62
Hospitalisation mania/depression	0.6 ± 1.00	0.6 ± 1.00	0.69
MADRS	7.5 ± 8.2	6.1 ± 9.5	0.65
HAMD-21	6.9 ± 6.5	3.1 ± 3.3	0.02
HAMD-17	5.3 ± 5.0	2.6 ± 3.3	0.06
CGI Mania	1.8 ± 0.9	1.5 ± 0.8	0.27
CGI depression	2.1 ± 1.5	1.4 ± 0.6	0.07
CGI bipolar	2.0 ± 1.1	1.7 ± 0.8	0.42
YMRS	2.2 ± 1.8	1.9 ± 2.6	0.72

The psychotropic medication taken by the bipolar patients in the omega-3 group and in the corn oil group is shown in Table 2. With few exceptions with regard to the dose of the medication prescribed, equally distributed between treatment groups, psychotropic medication was stable during the study period.

**Table 2:** Psychotropic medication

	Omega-3 (n = 23)	Corn oil (n = 19)
Quetiapine	n = 13	n = 13
Olanzapine	n = 4	n = 1
Risperidone	n = 1	n = 0
Haloperidol	n = 0	n = 1
Aripiprazole	n = 2	n = 1
Melperone	n = 1	n = 0
Prothipendyl	n = 1	n = 0
Lithium	n = 5	n = 5
Valproate	n = 4	n = 7
Lamotrigine	n = 4	n = 9
Lorazepam	n = 2	n = 1
Diazepam	n = 1	n = 0
Zopiclone	n = 3	n = 0
Venlafaxine	n = 0	n = 4
Mirtazapine	n = 0	n = 1
Escitalopram	n = 0	n = 2
Sertraline	n = 1	n = 0
Fluoxetine	n = 1	n = 0
Trimipramine	n = 0	n = 1
Agomelatine	n = 1	n = 0
Citalopram	n = 1	n = 0
Duloxetine	n = 1	n = 0
Doxepin	n = 1	n = 0
Pregabalin	n = 1	n = 0
No medication	n = 2	n = 0
No data	n = 3	n = 0

### Standardized rating scales

At baseline there was a significant difference in terms of the total score of the 21-item HAMD scale. According to this scale, patients in the omega-3 group were more depressed (6.9 ± 6.50) than those in the corn oil group (3.1 ± 3.25) (p = 0.019), though still not meeting the criteria for a depressive episode.

At end of study, in none of the standardized rating scales a significant difference between patients in the omega-3-group and the corn oil group was found. Endpoint and baseline scores were not significantly different (Table 3).

**Table 3:** Standardized rating scales endpoint and change from baseline, respectively: mean/median (SD) [missing values]

	Omega-3 (n = 23)	Corn oil (n = 19)	p-value
MADRS LOCF	6.06|0 (9.53)	7.45|5 (8.24)	0.6485
MADRS change	3.2|0 (6.5)	2.22|0.5 (8.09)	0.7031
HAMD LOCF	5.56|2 (7.7)	6.45|5.5 (6.16)	0.7105
HAMD change	2.44|0.5 (5.11)	− 0.16|0 (5.44)	0.1558
HAMD-17 LOCF	4.25|1 (6.77)	5.25|3 (5.24)	0.6308
HAMD-17 change	1.69|0 (4.69)	0.21|− 1 (4.96)	0.3726
CGI MANIA LOCF	1.27|1 (0.59)	1.35|1 (0.81)	0.7282
CGI MANIA change	− 0.36|0 (0.74)	− 0.44|0 (1.04)	0.7843
CGI depression LOCF	1.67|1 (1.11)	2.1|1.5 (1.33)	0.3031
CGI depression change	0.21|0 (0.89)	0.11|0 (1.02)	0.7631
CGI bipolar LOCF	1.67|1 (1.11)	2.05|2 (1.1)	0.3187
CGI Bipolar change	− 0.14|0 (1.23)	0.11|0 (0.83)	0.5144
YMRS LOCF	2.06|0 (3.82)	1.2|1 (1.54)	0.4063
YMRS change	0.12|0 (3.4)	− 0.94|− 1 (1.59)	0.2631

### SDNN

At baseline mean SDNN in patients in the omega-3 group was 34.4 ± 13.30 ms, and in the corn oil group 32.2 ± 16.65 ms (n.s.) (Table 4). At endpoint mean SDNN in patients in the omega-3 group was 39.8 ± 12.25 ms, and in the corn oil group 33.89 ± 17.24 (n.s.) (Table 5). The change in SDNN (value at endpoint minus value at baseline) was 1.8 ± 14.35 ms in the corn oil group and 5.4 ± 18.19 ms. in the omega-3 group. There was no significant difference in the change of the SDNN (in comparison of baseline and endpoint) between groups (Table 6). In addition, no correlation between changes in SDNN and change in the omega-3 index in the omega-3 group was detected (correlation coefficient = 0.02, p = 0.94). The same was true for the corn oil group (correlation coefficient =  − 0.11, p = 0.91).

**Table 4:** Baseline values: mean/median (SD)

	Omega-3 (n = 23)	Corn oil (n = 19)	p-value
SDNN (ms)	34.4|32.1 (13.30)	32.2|28.3 (16.65)	0.45
LF (ms^2^)	0.23|0.18 (0.17)	0.3|0.28 (0.22)	0.2379
HF (ms^2^)	0.11|0.11 (0.07)	0.14|0.08 (0.13)	0.3873
LF/HF ratio	2.84|1.96 (2.61)	2.2|2.02 (1.19)	0.2995
Omega-3 index (%)	4.7|4.8 (0.69)	4.6|4.8 (1.35)	0.67

**Table 5:** Endpoint values: mean/median (SD)

	Omega-3 (n = 23)	Corn oil (n = 19)	p-value
SDNN (ms)	39.8|40.27 (12.25)	33.89|31.55 (17.24)	0.21
LF (ms^2^)	0.43|0.24 (0.43)	0.39|0.16 (0.5)	0.8046
HF (ms^2^)	0.16|0.14 (0.13)	0.16|0.06 (0.24)	0.9625
LF/HF ratio	4.38|2.76 (4.31)	3.08|2.89 (1.58)	0.1871
Omega-3 Index (%)	9.69|9.96 (2.35)	4.23|4.13 (0.87)	< 0.0001

**Table 6:** Change endpoint vs. baseline: mean/median (SD)

	Omega-3 (n = 23)	Corn oil (n = 19)	p-value
SDNN (ms)	5.4|5.8 (18.19)	1.8|0.0 (14.35)	0.22
LF (ms^2^)	0.2|0.0 (0.42)	0.1|− 0.0 (0.22)	0.19
HF (ms^2^)	0.0|0.0 (0.14)	0.0|− 0.0 (0.04)	0.34
LF/HF ratio	1.5|0.8 (2.31)	0.9|0.6 (1.74)	0.84
Omega-3 Index (%)	5.0|5.3 (1.94)	− 0.4|− 0.3 (1.06)	< 0.0001

Linear mixed models with random intercept were created, initially unadjusted (Tables 7, 8), then adjusted for age and gender (Tables 9, 10). Unadjusted as well as adjusted for age and gender no significant effect of group affiliation (unadjusted: p = 0.5873, adjusted: p = 0.8270) and the omega-3 index (unadjusted: p = 0.8143, adjusted: p = 0.8377) on SDNN were observed.

**Table 7:** Effect of Omega-3 Index on SDNN, unadjusted

	95% CI	p-value
Intercept	10.698–59.239	0.0032
Omega-3 index	− 5.453–4.735	0.8143
Time	− 27.714–24.799	0.8161
Omega-3 × time	− 4.389–6.206	0.6382

**Table 8:** Effect of group on SDNN, unadjusted

	95% CI	p-value
Intercept	26.469–38.012	0.0000
Group verum	− 5.841–9.783	0.5873
Time	− 6.225–9.587	0.6114
Group verum x time	7.122–14.500	0.4422

**Table 9:** Effect of Omega-3 Index on SDNN, adjusted for age and sex

	95% CI	p-value
Intercept	0.0490–67.3953	0.0419
Age	− 0.2779–0.2875	0.9953
Gender	− 8.3393–3.5283	0.4163
Omega-3 index	− 5.4301–6.3270	0.8377
Time	− 27.1000–33.2260	0.8107
Omega-3 × time	− 6.0341–6.0408	0.9955

**Table 10:** Effect of group on SDNN, adjusted for age and sex

	95% CI	p-value
Intercept	22.0956–56.2707	0.0001
Age	− 0.3337–0.2395	0.7813
Gender	− 9.2594–2.6153	0.4067
Group Verum	− 7.4779–8.8112	0.8270
Time	− 6.6198–10.0832	0.6169
Group Verum × time	− 7.1723–15.5608	0.3089

In addition, linear models on the SDNN change were calculated including group affiliation and change of omega-3 index as predictors. Neither for group (unadjusted: p = 0.4445, adjusted: p = 0.4447) nor for change of omega-3 index (unadjusted: p = 0.6462, adjusted: p = 0.6241) statistically significant effects were found.

However, in the intervention group, but not in the control group, the result of the regression model, with explaining variables EPA change, DHA change and omega-3 index at baseline and age, gender and diagnosis of bipolar disorder as further co-variables in the model, indicates a positive association of the omega-3 index at baseline with an increase of SDNN during the study (p = 0.04). In addition, the change of DHA shows a positive association with concurrent change of SDNN in the study (i.e. increasing the concentration of DHA goes along with increasing SDNN, (p = 0.01), while the change of EPA shows a negative association with SDNN (i.e. increasing concentration of EPA goes along with decreasing SDNN, p = 0.01).

### LF, HF und LF/HF

In terms of the absolute values of HF, LF, and the LF/HF ratio, no significant differences between the corn oil and the omega-3 group were found at baseline and at the end of the study period (Tables 4, 5). Linear mixed models were created including group affiliation and omega-3 index as predictors. Neither for group (LF p = 0.5535, HF p = 0.4579, LF/HF ratio p = 0.4654) nor for omega-3 index (LF p = 0.3810, HF p = 0.7065, LF/HF ratio p = 0.5564) significant influence on frequency parameters were shown.

As a next step the change of LF, HF, LF/HF ratio (value at endpoint minus value at baseline) was illustrated. Using Wilcoxon signed-rank test no significant differences between the corn oil and the omega-3 group regarding the change of LF (p = 0.19), HF (p = 0.34) and LF/HF ratio (p = 0.84) were demonstrated (Table 6).

Linear models on the change of LF, HF, LF/HF ratio were calculated including group affiliation and change of omega-3 index as predictors, initially unadjusted, then adjusted for age and gender. Neither for group (unadjusted: LF p = 0.53, HF p = 0.54, LF/HF ratio p = 0.39. adjusted: LF p = 0.76, HF p = 0.66, LF/HF ratio p = 0.57) nor for change of omega-3 index (unadjusted: LF p = 0.47, HF p = 0.50, LF/HF ratio p = 0.63; adjusted: LF p = 0.49, HF p = 0.47, LF/HF ratio p = 0.63) statistically significant effects on any of these variables was found.

### Omega-3-index

The mean omega-3 index at baseline was 4.6 ± 1.35% in the corn oil group, compared to 4.7 ± 0.69% in the omega-3 group (n.s.), (Table 4), the mean omega-3 index at endpoint was 4.23 ± 0.87% in the corn oil group, compared to 9.69 ± 2.35% in the omega-3 group (p < 0.0001) (Table 5). The change of omega-3 index after 12 weeks (value at endpoint minus value at baseline) was − 0.4 ± 1.06% in the corn oil-group, compared to 5.0 ± 1.94% in the omega-3 group (p =  < 0 0.0001) (Table 6).

### Mood ratings, new episodes

In the omega-3 group, 3 patients experienced a depressive episode, but none in the placebo group. There were no significant differences in change from baseline to end point in any of the standardized rating scales (Table 3).

## Discussion

In our randomized, controlled intervention trial, comparing the effects of 2120 mg EPA plus 600 mg DHA per day with a corn oil placebo in euthymic bipolar patients with a low omega-3 index and reduced heart rate variability no significant effect of omega-3 fatty acids on SDNN or frequency-domain measures HF, LF and LF/HF ratio could be detected. In light of the positive effects of omega-3 fatty acids on parameters of HRV in cardiovascular patients (Harris et al. 2006) this is a perplexing finding.

Was our trial inadequately designed or conducted to detect an effect? As discussed in the introduction, our trial had a high likelihood of detecting a beneficial effect of EPA and DHA on HRV in bipolar patients. By selecting bipolar patients with low baseline levels of EPA and DHA, and with a low SDNN, we selected a population for our trial likely to benefit from our intervention. The trial design we used has been suggested for all trials with omega-3 fatty acids with cardiovascular endpoints (Rice et al. 2014). Our trial conforms the Guidelines for the Design, Conduct, and Reporting of Human Intervention Studies to Evaluate Health Benefits of Foods, and, inadvertently, the recent Guidelines for Reporting Articles on Psychiatry and Heart Rate variability (Quintana et al. 2016). Also, we fulfilled our case estimate in the verum group, although not completely in the placebo group (Table 1). Of note, however, changes observed in parameters of HRV were minimal (Tables 4–6). The fact that no correlation between changes in SDNN and change in the omega-3 index could be detected, neither in the intervention group nor in the control group, also argues against the fact that an increase in the number of study participants would have led to a significantly different result. Our trial was similar in length, when compared to positive trials in other patient populations (Rovere and Christensen 2015). Taken together, we feel that our trial was adequately designed and conducted to detect an effect of omega-3 fatty acids on HRV. In the intervention group the increase in the concentration of DHA was accompanied by an increase in SDNN, while for EPA it was the opposite. Therefore, would it have been possibly advisable to increase the DHA content of the study medication to improve heart rate variability? Few data exist regarding differential effects of EPA and DHA on heart rate variability (Xin et al. 2013; Innes and Calder 2020). Furthermore it is hard to predict the precise consequences of a modification of the DHA content of the omega-3 fatty acids supplement on membrane fatty acid composition (Harris et al. 2021; Pal et al. 2020; von Schacky 2019). In addition recent evidence suggests that high dose pure EPA has advantages compared to a high-dose combination of EPA/DHA regarding cardiovascular risk reduction in patients at increased cardiovascular risk (Bhatt et al. 2019; Nicholls et al. 2020). Therefore increasing the DHA content of the study medication wouldn’t probably have changed the outcome of the trial.

Did we measure HRV adequately, and were the parameters of HRV measured appropriate? Technically, the method we used fulfills current criteria, and we also took special care to minimize confounders (Quintana et al. 2016). Our primary endpoint was SDNN, and we also measured LF, HF and the LF/HF ratio. According to recent (systematic) reviews, these parameters are informative, and differ from healthy controls in bipolar patients (Bassett 2016; Faurholt-Jepsen et al. 2017). SDNN is thought to reflect predominately (but not exclusively) sympathetic activity, while HF is indicative of parasympathetic activity, with LF reflecting a mix of both (Alvares et al. 2016; Bassett 2016). Therefore, we feel that little additional information could be gleaned from additionally studying root mean square of normal to normal interval differences (RMSSD) and the proportion of RRIs that differ more than 50 ms (pNN50) (Alvares et al. 2016; Bassett 2016). Taken together, we feel that we measured appropriate parameters of HRV and performed the measurement adequately.

We analyzed erythrocyte fatty acids, that have a low biological variability, with a method of analysis that not only has a low analytical variability, but also the largest database of all methods of fatty acid analysis (von Schacky 2012; 2015, 2018). Moreover, in the intervention group, the omega-3 index increased from a mean (± SD) of 4.7 (0. 69) to 9.69 (2.35), while it remained constant in the placebo group; SD’s were small; together indicating excellent compliance with both trial regimens (Tables 4, 5). Therefore we think that we achieved our objective to substantially change omega-3 status in the intervention group, to bring the intervention group into the proposed target range for the omega-3 index of 8–11%, and to generate a large difference in omega-3 status between verum and placebo groups. Furthermore, as noted earlier, we did not find a significant correlation between changes in SDNN and change in the omega-3 index, neither in the omega-3 group, nor in the corn oil group (data not shown). This latter result much resembles the results of our baseline study, in which we did not find any (positive) relationship between omega-3 status of our trial participants and the parameters of HRV measured. Our findings also support our use of corn oil as a placebo (Tables 4–6).

Is the result due to confounders? While our knowledge of the effects of the various psychotropic drugs on measures of HRV is still limited, in particular for lithium, lamotrigine and valproic acid (Tomson et al. 1998) the data so far suggest that psychotropic drugs, including our currently used antidepressants (Kemp et al. 2010; Licht et al. 2008; O'Regan et al. 2015) as well as quetiapine (Huang et al. 2016) appear to have a significant negative effect on measures of heart rate variability (Alvares et al. 2016). Although use of psychotropic medication was evenly distributed between verum and placebo groups, therefore excluding a systematic bias (Table 2) one might speculate whether these psychotropic drugs may not only have a negative impact on parameters of heart rate variability on their own but may also prevent omega-3 fatty acids from improving parameters of HRV (Carney et al. 2010) in the way they usually do. This hypothesis is backed by two lines of evidence. In the first place in the trial our study best compares with in terms of patient population (affective disorders), dose (930 mg of EPA and 750 mg of DHA), and length of intervention (10 weeks) determination of omega-3 fatty acids status (i.e. omega-3 index), use of psychotropic medication (sertraline, an antidepressant) as well as type of placebo (i.e. corn oil) (Carney et al. 2010) there was a significant treatment X time interaction for the primary measure of HRV, in very low frequency [VLF (p = 0.009)], and for heart rate (HR (p = 0.03)). However, the interactions for all secondary HRV indices were not significant [in HF (p = 0.12), in LF (p = 0.11), in ultra low frequency (ULF (p = 0.23))]. SDNN was not measured. In the second place, in our study, in the intervention group, but not in the control group, there was a positive association of the omega-3 index at baseline with an increase of SDNN during the study (p = 0.04).This may indicate that in the presence of psychotropic drugs such as antidepressants or quetiapine a higher omega-3 index at baseline (or possibly a larger dose of omega-3 fatty acids) is needed to bring about a significant increase in SDNN in the intervention group. Interestingly, in the afore mentioned trial our study best compares with (Carney et al. 2009, 2010), in the intervention but not in the control group, baseline red blood cells (RBC) levels of EPA + DHA were significantly higher among those whose depression subsequently remitted compared with those whose depression did not remit (Carney et al. 2016) while there was no significant difference between the treatment groups as a whole in the study in question (Carney et al. 2009) and a more recent trial (Carney et al. 2019). This suggests that a similar mechanism might exist regarding the antidepressant effects of omega-3 fatty acids in the presence of psychotropic drugs such as antidepressants (Guu et al. 2019).

It has recently been suggested that omega-3 fatty acids increase HRV via alterations in intrinsic pacemaker rate rather than via changes in cardiac autonomic neural regulation (Billman 2013). This would be in keeping with an earlier observation in patients with a cardiac transplant, a situation with no or little parasympathetic control of cardiac rhythm. Dietary omega-3 fatty acids appeared to alter electrophysiological properties of the heart itself (Harris et al. 2006). Bipolar disorder has a genetic component, with a striking number of the calcium channel gene superfamily being involved, among many other genes (Xin et al. 2013; Zelniker et al. 2021). Taken together, one might speculate, that in bipolar disorder, a genetically defined variant of a calcium channel of the intrinsic cardiac pacemaker might be resistant to the effects of EPA and DHA. Clearly, however, this speculation needs to be substantiated by further research.

This study could not detect any significant difference between number of new affective episodes or change in mood ratings between the study groups. This is not surprising given that the study was not powered to detect such changes—and the current evidence (McPhilemy et al. 2020). The numerically higher number of mood episodes in the omega-3 group might be a result of patients in the omega-3 group being more depressed at baseline.

## Conclusions

In our randomized, controlled intervention trial, comparing the effects of 2120 mg EPA plus 600 mg DHA per day with a corn oil placebo in euthymic bipolar patients with a low omega-3 index and reduced heart rate variability no significant effect of omega-3 fatty acids on SDNN or frequency-domain measures HF, LF and LF/HF ratio could be detected. Given the positive evidence of omega-3 fatty acids on parameters of HRV in cardiovascular patients this was an unexpected finding with, among others, the effect of psychotropic medication present in our trial or the genetics of bipolar disorder itself being possible culprits. Clearly further research is urgently needed to better understand the underlying mechanisms.

## Data Availability

The datasets used and/or analysed during the current study are available from the corresponding author on reasonable request.

## Abbreviations

BDI: Beck Depression Inventory
CGI-BP: Clinical Global Impressions Scale for Bipolar Illness
DHA: Docosahexaenoic acid
DSM-IV: Statistical Manual of Mental Disorders Fourth Edition
ECG: Electrocardiogram
EPA: Eicosapentaenoic acid
HAMD: Hamilton Rating Scale for Depression
HF: High frequency
HFP: High frequency power
HR: Heart rate
HRV: Heart rate variability
LF: Low frequency
LF/HF ratio: A ratio of low frequency to high frequency
LFP: Low frequency power
MADRS: Montgomery- Åsberg Depression Rating Scale
pNN50: Proportion of RRIs that differ more than 50 ms
RBC: Red blood cells
RMSSD: Root mean square of normal to normal interval differences
SCID: Structured Clinical Interview
SDNN: Standard deviation of the normal-to-normal interval
ULF: Ultra low frequency
VLF: Very low frequency
YMRS: Young Mania Rating Scale
